# In Vitro Comparative Analysis of Fracture Resistance in Inlay Restoration Prepared with CAD-CAM and Different Systems in the Primary Teeth

**DOI:** 10.1155/2016/4292761

**Published:** 2016-10-17

**Authors:** Huseyin Simsek, Sera Derelioglu

**Affiliations:** ^1^Department of Pedodontics, Faculty of Dentistry, Ordu University, 52100 Ordu, Turkey; ^2^Department of Pedodontics, Faculty of Dentistry, Ataturk University, 25030 Erzurum, Turkey

## Abstract

*Objective*. The aim of this study was to compare to fracture resistance test of inlay restorations prepared using direct inlay technique (Gradia® Direct Composite) and Indirect Restoration System® (Gradia Indirect Composite) and CAD/CAD system (Vita Enamic® Block).* Study Design*. 48 noncarious extracted maxillary second primary molars were randomly divided into 4 groups with 12 in each group. All the teeth were prepared based on inlay class II preparations except for the control group. Other groups were restored with Gradia Direct Composite, Gradia Indirect Composite, and Vita Enamic Block, respectively. All restorations were cemented self-adhesive dual cure resin (3M Espe, RelyX™ Unicem Aplicap). A fracture test was performed using a compressive load. Results were analyzed using one-way analysis of variance and Duncan's post hoc multiple comparison tests (*α* = 0.05).* Results*. Vita Enamic Block and Gradia Indirect Composite showed significantly higher fracture resistance than Gradia Direct Composite (*p* < 0.05). There was no significant difference fracture resistance between Vita Enamic Block and Gradia Indirect Composite (*p* > 0.05). All restorations tested led to a significant reduction in fracture resistance (*p* < 0.05).* Conclusion.* In inlay restorations, Indirect Restoration Systems and CAD/CAM systems were applied successfully together with the self-adhesive dual cure resin cements in primary molars.

## 1. Introduction

It is very difficult to make restoration in children in the existence of extensive coronal destruction of primary molars [1]. There are some disadvantages of stainless steel crowns such as their nonaesthetic appearance and possibility of damaging gingival tissues. Likewise, the direct restorations require extensive working time, creating operation difficulty in the noncooperative children and failures due to polymerization shrinkage [2, 3]. When all of these problems are taken into consideration, there has been a need for the treatment options that can be easily prepared and applied and can meet the aesthetic needs.

It is possible to obtain successful treatment results by applying inlay restorations in children through the use of different systems. The ways of making inlay restorations were first introduced by Dr. Philbrook in 1897. These restorations which are extraorally made and cemented into the teeth are classified as inlays, onlays, and overlays depending on the dimensions of the cavities [4]. It is possible to make the inlay restorations with both composite resins and ceramics. With the development of the restoration materials, adhesive cement, and systems that are being used, making restorations became easier and has become more attractive for children [5].

With the evolving technology, inlay restorations can be easily fabricated with chair-side CAD/CAM systems by using blocks with a variety of alternative shades [5, 6]. Likewise, the manufacturers have specified that better polymerization, improvement in the physical properties of the restorations, and chance of easier work could be provided in the composite inlays prepared with the recently developed Gradia Indirect Restoration System (GC Gradia Indirect Composite, Labolight LV-III) [7]. Researched with these new developed systems, studies for the primary teeth have been limited only to the case reports. Therefore, the aim of this study was to determine the superiority prepared with direct inlay technique, indirect composite systems, and CAD/CAM systems in the primary teeth in terms of their usage and fracture resistance.

## 2. Materials and Methods

### 2.1. Preparation of the Teeth Samples

48 noncarious extracted maxillary second primary molars were removed from the soft tissue deposits with a hand scalar. The teeth were disinfected in 10% thymol for 24 hours and then stored in distilled water at 4°C until preparation. Then, the teeth specimens were embedded perpendicularly in the self-cured acrylic resin (Takilon®, WP-Dental, Barmstedt, Germany) up to 2 mm below the enamel-cement junction in the standard plastic molds. All of the teeth were randomly divided into 4 groups with 12 in each group. The procedures in this study were approved by the Ethics Committee of Ataturk University Faculty of Dentistry (Erzurum/TURKEY).

### 2.2. Cavity Preparation

**Figure 1:**
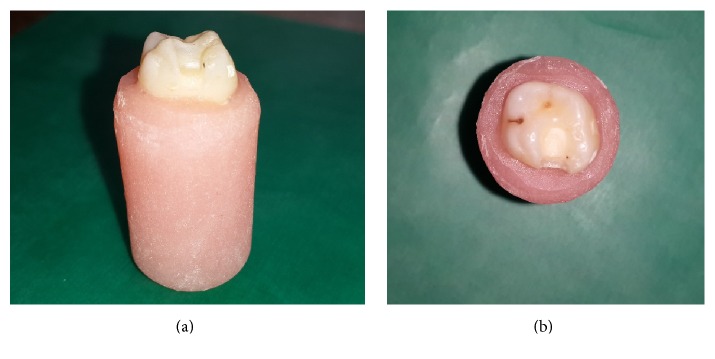
Proximal view of inlay class II preparations (a); occlusal view of inlay class II preparations (b).

All the teeth were prepared based on their inlay class II preparations except for the control group. Inlay preparations were prepared using diamond trunk conic burs (KG Sorensen, Barueri, SP, Brazil). The gingival wall was prepared 0.5 mm above the enamel-cement junction, while the buccolingual width was prepared as 2.5 mm. Occlusal depth was designed as 2.5 mm below the enamel-dentine border (Figure 1). Millimetric end of the periodontal probe was used for measuring the cavities. The angle between the cavity floor and the sidewalls was observed to be 95–100 degrees expanding towards the occlusion.

### 2.3. Preparation of the Restorations

*First Group (Control)*. No preparation was performed.

*Second Group*. The teeth were restored by using direct inlay technique. Firstly, separating liquid (GC Gradia separator) was applied to the cavity. Gradia Direct Posterior Composite (GC America Inc., Alsip, IL, USA) was placed in the cavity in two layers and was removed from the cavity after the prepolymerization of 10 seconds. Afterwards, it was light-cured for 3 minutes in total from all surfaces with a light-emitting diode (LED) polymerizing unit (Elipar S10; 3M ESPE, St. Paul, MN) with irradiance of 1200 mW/cm^2^.

**Figure 2:**
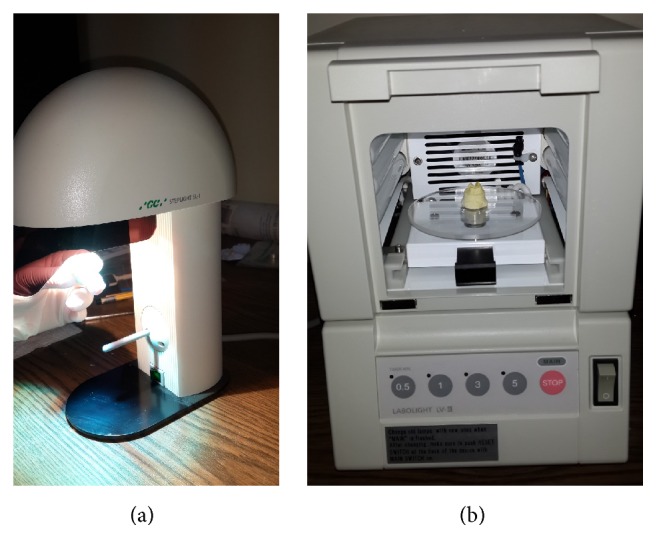
Prepolymerization with the Steplight SL-1 (a), final polymerization with Labolight LV-III (b).

*Third Group*. The teeth were restored by using Gradia Indirect Restoration System (GC America Inc., Alsip, IL, USA). The prepared teeth were impressed with a polyvinylsiloxane material (Elite HD, Zhermack, Rovigo, Italy) and impression was poured with type IV die stone (Elite Rock, Zhermack, Rovigo, Italy). All stone dies were sealed with a die hardener (Gradia Die Hardener) and then a thin layer of separator (Gradia separator) was applied for isolation. Gradia Indirect Composites in the system were placed in the cavity in two layers and each layer was exposed to prepolymerization with the Steplight SL-1 (GC America Inc., Alsip, IL, USA) for 10 seconds (Figure 2(a)). Finally, the final polymerization was performed for 3 minutes with the Gradia Labolight LV-III (Figure 2(b)). 

**Figure 3:**
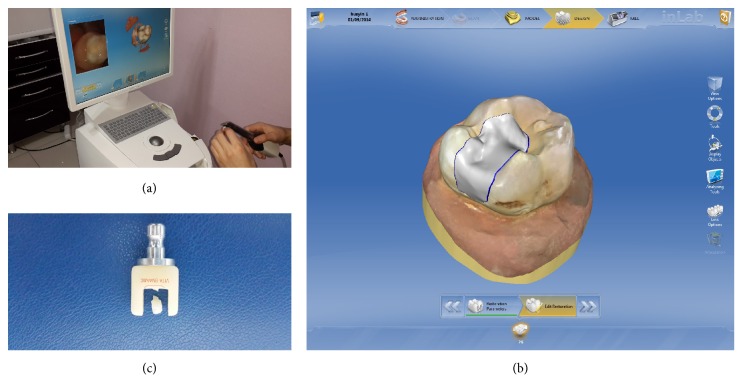
Prepared teeth scanning with CEREC Omnicam camera (a), restoration designing on virtual model (b), and milled Vita Enamic Block (c).

*Fourth Group*. The teeth were restored by using CAD/CAM system (Sirona, Bensheim, Germany) and Vita Enamic Blocks (Vita Zahnfabrik, Bad Sackingen, Germany). The prepared teeth were scanned with CEREC Omnicam camera and a color 3D virtual model was obtained (Figure 3(a)). The restoration design was made on the virtual model (Figure 3(b)). Vita Enamic Blocks were placed in the milling unit (CEREC^®^ MC XL) and the blocks were milling according to the design (Figure 3(c)).

### 2.4. Cementation of the Restorations

The cementation of the inlay restorations was performed with self-adhesive resin cement (3M Espe, RelyX Unicem Aplicap). Cementation was done according to the manufacturers' instructions for use. The inner surface of the composite inlays (Gradia Direct Composite and Gradia Indirect Composite) were sandblasted for 10 seconds with aluminum oxide particles and the inner surface of the blocks were etched with 10% hydrofluoric acid for 60 seconds and rinsed with water for 30 sec and dried for 20 sec. Silane was applied to inner surfaces of all restorations (Ultradent® Silane, Ultradent Products Inc., UT, USA) and after 60 seconds of waiting, air was sprayed to spread it homogenously. All of the samples were cemented using RelyX Unicem Aplicap. Afterwards, all surfaces including occlusal, buccal, lingual, and proximal were cured for 40 seconds for each. After the polymerization, the polishing was performed with the polishing discs (Sof-Lex™, 3M Espe, USA).

### 2.5. Fracture Test

The fracture resistance of the teeth was measured using Instron® universal testing machine (Instron, Model 2710-003, Instron Corp., USA). The stainless steel bar with rounded ends of 3.5 mm diameter was used. The steel bar was adjusted to simultaneously contact the buccal and palatal cusps of all restorations during the fracture test. Pressure was performed with 0.5 mm/min of cross-head speed and the data obtained as a result of the fracture test were calculated in Newtons.

The Shapiro-Wilks normality test was applied to the fracture test data. Because the data show a normal distribution, results were analyzed with statistical software (SPSS version 19.0, SPSS Inc., Chicago IL, USA) using a one-way analysis of variance (ANOVA) and Duncan's post hoc multiple comparison tests (*α* = 0.05).

## 3. Results

**Figure 4:**
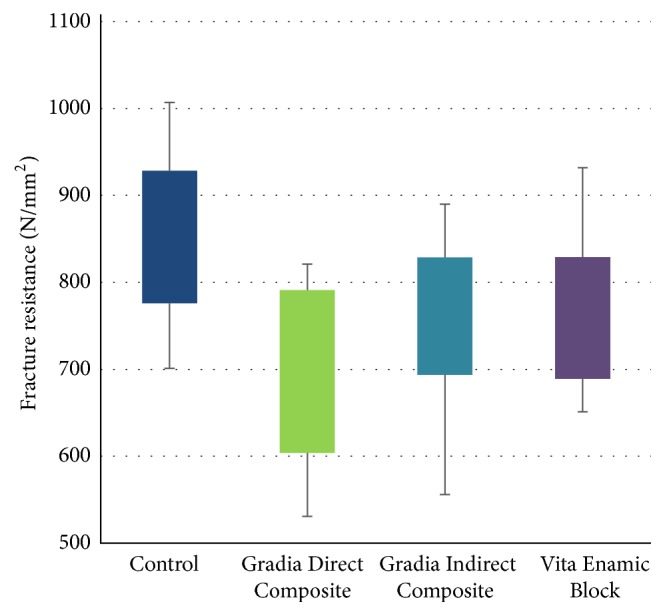
Descriptive statistics of fracture resistance of test groups in this study.

The fracture resistance means in all groups are shown in Table 1. When the fracture resistance values of the groups are compared, it was seen that the Gradia Indirect group has the lowest mean fracture resistance (677.7 ± 105.4) while the control group has the highest value (850.2 ± 96.2). It was also seen that the fracture resistances of Gradia Indirect Composite and Vita Enamic Blocks were statistically similar (resp., 764.8 + 94.0 and 762.3 + 95.3) (Figure 4).

According to the results of fracture resistance testing, a statistically significant difference was found among the groups (*p* = 0.001). Duncan's post hoc test was used to determine which group results are significantly different from others. According to the results of this analysis

(i) the control group with the highest fracture resistance has a significant difference from other groups (*p* < 0.05);
(ii) Gradia Direct group with the lowest fracture resistance is significantly different from other groups (*p* < 0.05);
(iii) no statistically significant difference has been found between Vita Enamic Blocks and Gradia Indirect groups with very similar values (*p* > 0.05).

## 4. Discussion

ln pediatric dentistry, time is more important than in the other areas of dentistry. In the restoration of the primary teeth which have more extensive loss in the younger patient, the placement of direct composite resins with the incremental technique and curing each increment lengthens the treatment a lot and this might cause this child patient to get bored [8], because the placement of direct resin composite requires technical sensitivity, patient cooperation, better isolation of the site, and an extensive working time. In addition, when the child gets tired and closes his mouth frequently, it causes salivary contamination and therefore the reduction of the restoration success [9].

Recently, ideal contact points, good surface finishing, obtaining more aesthetic and more longevity restorations, and better marginal adaptation were becoming popular in indirect restorations [10, 11]. One of the indirect restoration techniques is inlay restorations. Inlay restorations protect the tooth structure very well and may be an ideal alternative to the crown restorations [12]. Due to the increase of aesthetic expectation in pediatric dentistry, the need for different restoration techniques and the spreading of ceramic and composite materials made the ceramic and composite inlays outstanding in this area. When the indirect inlay techniques are compared with direct composite techniques, there is placement of the tooth with an anatomy close to the natural one, better control of the occlusal and proximal contact points, better marginal integrity especially in the gingival wall, minimal polymerization shrinkage caused by adhesive agent, better polishing and finishing opportunities, shorter clinical work, and less contamination risk [13].

Indirect Restoration Systems and the new system of CAD/CAM practices have been started to be used in indirect restorations in pediatric dentistry; and it has been reported that this method can be prepared and cemented in a shorter time than the placement time of the SSCs and it can be an alternative to the SSCs in terms of patient satisfaction, endurance, abrasion properties similar to the enamel, and a better quality of the marginal adaptation [14, 15]. In addition, reduction of the errors caused by the conventional impression methods thanks to the screening method in CAD/CAM applications and the presence of a program providing the preplanning of the restoration as well as the production of the restoration without the need for a laboratory makes it possible to have CAD/CAM restorations as the future restorations in pediatric dentistry [16]. Despite all of these specified advantages, the clinical data regarding CAD/CAM applications in pediatric dentistry are limited to the case reports. Therefore, in order to test the applicability of the CAD/CAM restorations in pediatric dentistry, we compared it with the other commonly used methods by working in primarily in vitro study.

It is of critical importance to minimize the unconverted (co)monomers amount in resin based materials and to polymerize them at the right light source for a sufficient time in order to prevent damage to the biological tissues [17]. Unfortunately it is not always possible to completely perform polymerization in the child patients [18]. We have aimed for inlay techniques in our study.

Self-adhesive dual cure resin cement (RelyX Unicem Aplicap) was used in our study. Self-adhesive dual cure resin cement can be applied in a short time without the need for any preprocessing such as primer, adhesive, and acid. Moreover, this material has less technical sensitivity and fluoride releasing, so it can be used more attractive for dentists.

Depending on both forces occurring in occlusion and function in the used restorative materials, stress might be formed within the material. The fracture resistance is an important criteria in evaluating the long-term success of restorative materials [19, 20]. Although fracture as a result of normal bite forces in healthy teeth is observed rarely, fracture is observed more frequently in the teeth with cavity preparation or caries. As a result of cavity preparation, the structure of the tooth is weakened and its tendency towards fracture is increasing. It was seen in a study that the structure of the upper premolar teeth with a MOD cavity weakened by 59% [21]. Therefore, it was found that the teeth in the control group which did not undergo any restoration process had the highest fracture values in our study.

It was observed that the fracture resistance of Gradia Indirect Composites (Gradia Indirect Restoration System) was more than the Gradia Direct Composites. The reason for this was that the filing rate within the Gradia Indirect Composites was increased and the physical properties were improved thanks to the silane coated ceramic particles. In addition, the light curing units used during final polymerization and prepolymerization (Labolight LV-III, Steplight SL-I) provided homogeneous polymerization in the low wavelength and the polymerization shrinkage was reduced.

The fracture resistance values of Vita Enamic Blocks and Gradia Indirect Composites were found to be similar. Vita Enamic Block is the first hybrid dental ceramic in the world with a dual-network structure. Vita Enamic is a dental hybrid material that combines the positive characteristics of a ceramic and a composite. Vita Enamic Blocks abrasion properties are similar to enamel, so it is reported that it protects better the antagonist teeth and enables a good adhesion with the self-adhesive dual cure resin cement due to its composite-like structure. The microstructural analyses showed a hybrid material composed of interconnected networks: a dominant ceramic and a polymer [22, 23]. High magnification microscopy showed a few microcracks in the network structure. These defects may decrease the mechanical properties of materials [24]. Despite its ceramic content, the dual network structure inside and its complete production under fabrication conditions increased the fracture resistance of Vita Enamic Blocks and its fracture resistance was found to be similar to the Gradia Indirect Composite.

## 5. Conclusions

The restorations prepared with indirect technique (Gradia Indirect Restoration System and CAD/CAM system) in case of more extensive loss of dental structure in the primary teeth can be preferred because of their better fracture resistance, aesthetic looks, implementation in a single visit, and shorter intraoral working-time. When all of these are considered, the restorations prepared with the indirect techniques in children and especially CAD/CAM restorations should be researched more in terms of in vivo and in vitro finding in terms of pediatric dentistry.

## Figures and Tables

**Table 1:** Mean and standard deviations of different experimental or control groups.

Groups	*N*	Mean	SD	Minimum	Maximum
Control	12	850.167^a^	96.238	701.0	1007.0
Gradia Direct Composite (direct inlay technique)	12	677.717^b^	105.465	531.2	821.2
Gradia Indirect Composite, (Indirect Restoration System)	12	764.817^c^	94.001	556.1	890.2
Vita Enamic Block (CAD/CAM system)	12	762.283^c^	95.321	651.5	932.1

Different small letter superscripts indicate that fracture resistance values are significantly different at *p* < 0.05.
